# Nocturnal Hypoxemic Burden Predicts Mortality in Patients Awaiting Heart Transplantation

**DOI:** 10.1111/jsr.70256

**Published:** 2025-11-27

**Authors:** Max Potratz, Tatevik Manukyan, René Schramm, Angelika Costard‐Jäckle, Uwe Fuchs, Michiel Morshuis, Sabina P. W. Guenther, Sebastian V. Rojas, Volker Rudolph, Jan F. Gummert, Henrik Fox

**Affiliations:** ^1^ Clinic for General and Interventional Cardiology/Angiology, Herz‐ und Diabeteszentrum NRW, Ruhr‐Universität Bochum Bad Oeynhausen Germany; ^2^ Division of Cardiology Northwestern University, Feinberg School of Medicine Chicago Illinois USA; ^3^ Clinic for Thoracic and Cardiovascular Surgery, Herz‐ und Diabeteszentrum NRW, Ruhr‐Universität Bochum Bad Oeynhausen Germany; ^4^ Clinic of Cardiac Surgery Ludwig Maximilian University (LMU) Munich Germany

**Keywords:** heart failure, heart transplantation, hypoxemic burden, hypoxia, mechanical circulatory support, mortality, sleep‐disordered breathing, survival

## Abstract

Risk stratification for patients with end‐stage heart failure (HF) awaiting heart transplantation (HTX) is crucial. Sleep‐disordered breathing (SDB) is a prevalent comorbidity, yet the impact of its resultant hypoxemic burden remains unclear in this population. We investigated the prognostic impact of nocturnal hypoxemic burden in patients with end‐stage HF awaiting HTX. We prospectively enrolled 101 patients with end‐stage HF listed for HTX at a single centre. Baseline polygraphy quantified nocturnal hypoxemic burden (time with SpO2 < 90%; t90). The primary endpoint was a 5‐year composite of all‐cause mortality or left ventricular assist device (LVAD) implantation. Multivariable Cox proportional hazards models were used for adjustment. During 5‐year follow‐up, 24 patients reached the primary endpoint. The primary finding was a significant, dose–response relationship between t90 (analysed as a continuous variable) and the primary endpoint (*p* < 0.001). In a multivariable Cox model adjusted for glomerular filtration rate (GFR) and left ventricular ejection fraction (LVEF), each 30‐min increase in t90 was associated with a 29% increase in hazard (Adjusted HR: 1.29, 95% CI: 1.14–1.47, *p* < 0.001). A t90 of ≥ 180 min was identified as a clinical threshold of high risk, conferring a > 5‐fold increase in hazard (Adjusted HR: 5.18, 95% CI: 1.69–15.81, *p* = 0.004). The cumulative duration of nocturnal hypoxemic burden is a powerful, independent predictor of death or LVAD implantation in patients with end‐stage HF. Routine assessment of hypoxemic burden should be considered for risk stratification in patients awaiting heart transplantation.

**Trial Registration:** ClinicalTrials.gov: NCT03026634

## Graphical abstract

Trial design and results on hypoxemic burden in the context of heart transplantation.

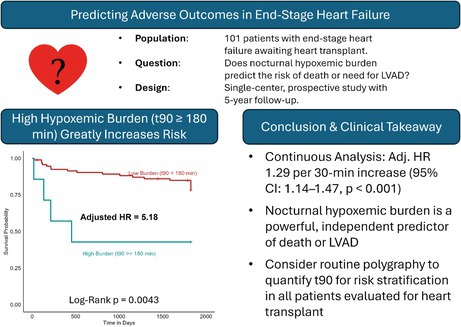

## 1 Introduction

Heart failure (HF) represents a growing global healthcare burden, characterised by high morbidity and mortality (McDonagh et al. 2021). Despite significant advances in guideline‐directed medical therapy, the management of HF is increasingly challenged by a rising number of patient comorbidities (Levy et al. 2002; Heidenreich et al. 2022; Sohns et al. 2023). For eligible patients with end‐stage disease, heart transplantation (HTX) remains the definitive treatment for improving long‐term survival (Sohns et al. 2021). However, outcomes after HTX are influenced by a variety of factors, with patient comorbidities playing an essential role (Sohns et al. 2021).

In this context, sleep‐disordered breathing (SDB) is a highly prevalent yet underdiagnosed comorbidity in the HF population (Fox et al. 2016). The pathophysiology of SDB is known to exert numerous deleterious effects on the cardiovascular system, including intermittent hypoxia, sympathetic nervous system overstimulation, and chronic inflammation, which can exacerbate underlying cardiac dysfunction (Sharma et al. 2021).

Traditionally, SDB has been quantified by the apnea‐hypopnea index (AHI) (Kapur et al. 2017). However, emerging evidence suggests that the cumulative hypoxemic burden, often measured as the total time spent with nocturnal oxygen saturation below 90% (t90) in minutes, may be a more robust and direct predictor of mortality than the AHI alone (Oldenburg et al. 2016). This is particularly relevant in HF, where nocturnal hypoxemia is directly related to increased hemodynamic stress (Gottlieb et al. 2009).

Despite the known prognostic importance of hypoxemic burden in other cardiovascular populations, its specific role in patients with end‐stage HF remains largely unexplored. SDB is rarely investigated as part of the routine clinical evaluation for patients on the HTX waiting list and is attended to even less in patients after they have received a transplant. This represents a critical gap in knowledge.

Therefore, this study was designed to investigate the impact of nocturnal hypoxemic burden on the 5‐year composite outcome of all‐cause mortality or LVAD implantation in patients evaluated for HTX.

## 2 Methods

### 2.1 Study Design and Patient Population

This single‐centre, prospective observational study was conducted at the Herz‐ und Diabeteszentrum NRW in Bad Oeynhausen, Germany. The study was approved by the local institutional review board (Reg. Nr. 48/2016), was ex ante prospectively registered at ClinicalTrials.gov (NCT03026634), and was conducted in accordance with the Declaration of Helsinki. All participants provided written informed consent.

Between January 2017 and May 2020, we screened 500 patients to finally enroll 101 consecutive patients with end‐stage HF referred for HTX evaluation. Key inclusion criteria were: (1) a diagnosis of HF with reduced ejection fraction (HFrEF) with a left ventricular ejection fraction (LVEF) of ≤ 35%; (2) New York Heart Association (NYHA) functional class III or IV symptoms; and (3) persistent symptoms despite optimised guideline‐directed medical therapy. Major exclusion criteria included active malignancy or other significant non‐cardiac conditions that would limit survival to less than 1 year.

### 2.2 Study Endpoints and Follow‐Up

As originally registered, this study was designed as a prospective, mechanistic, pre‐post study to assess the change in cardiorespiratory parameters 30 days after HTX. However, the collection of complete polygraphy data 30 days post‐transplantation proved logistically unfeasible for a significant portion of the cohort, precluding a formal analysis of the original pre‐post endpoints. Given the availability of a complete baseline dataset and robust long‐term follow‐up for the entire cohort, a deliberate decision was made to pivot the analysis to this prognostic cohort design: to investigate the power of *baseline* SDB metrics to predict long‐term adverse outcomes.

The primary composite endpoint for this analysis was all‐cause mortality or implantation of a durable left ventricular assist device (LVAD) within a 5‐year follow‐up period. Patient outcomes were prospectively tracked through routine visits to our outpatient HF clinic, telephone contact, and review of electronic health records. Follow‐up was complete for the entire cohort.

### 2.3 Cardiorespiratory Polygraphy

All patients underwent in‐hospital, unattended multichannel cardiorespiratory polygraphy (Embletta, Natus Medical Inc.) at the time of evaluation for HTX. An apnea was defined as a complete cessation of airflow for at least 10 s. A hypopnea was defined as a reduction in airflow of at least 30% for 10 s or more, accompanied by an oxygen desaturation of at least 3%. Respiratory events were classified as obstructive if associated with continued thoracoabdominal effort or central if both airflow and respiratory effort were absent (Kapur et al. 2017).

The overall severity of SDB was quantified using the AHI, calculated as the total number of apneas and hypopneas per hour of recording. SDB was diagnosed if the AHI was > 5 events/h. The SDB type was classified as predominantly central (CSA) or obstructive (OSA) if more than 50% of respiratory events were of the respective type (Kapur et al. 2017). The primary metric for nocturnal hypoxemic burden was the total time with an oxygen saturation below 90% (t90), measured in minutes. All studies were manually scored, and no patients received specific SDB treatment during the study period.

### 2.4 Statistical Analysis

Baseline characteristics of the study population are presented as mean ± standard deviation, median [interquartile range], or *n* (%) as appropriate. Group comparisons were performed using independent *t*‐tests, Mann–Whitney *U* tests, or chi‐square tests. Survival probabilities over the 5‐year follow‐up period were estimated using the Kaplan–Meier method, and curves were compared with the log‐rank test. To identify independent predictors of the endpoint, unadjusted and multivariable Cox proportional hazards regression models were built. The primary variable of interest, t90, was analysed both as a continuous variable (with its non‐linear relationship explored using restricted cubic splines) and as a dichotomized variable at a 180‐min threshold. Final multivariable models were adjusted for clinically significant confounders, including GFR and LVEF, and the proportional hazards assumption was verified for all models. A two‐tailed *p* value < 0.05 was considered statistically significant. All analyses were performed using Statistica software, version 14 (TIBCO Software Inc.) and R version 4.4.2 (R Foundation for Statistical Computing, Vienna, Austria) with the ‘survival’, ‘survminer’, ‘ggplot2’ and ‘rms’ packages.

## 3 Results

The final analysis included 101 patients with end‐stage HF actively listed for HTX. The primary endpoint of death or LVAD implantation within 5 years occurred in 24 patients. Baseline demographic, clinical, and sleep study characteristics, along with the complete univariate Cox regression analysis of the primary endpoint, are detailed in Table 1.

**TABLE 1:** Baseline patient characteristics and comparison by 5‐year outcome and univariate analysis.

	No endpoint reached	Death or LVAD implantation	*p*	Univariate analysis
	*n* = 77	*n* = 24		
Age (years)	48.7 ± 11.7	53.5 ± 9.6	0.069	HR 1.04 [0.99–1.09], *p* = 0.09
Gender (female)	58 (75.3)	18 (75)	0.975	HR 1.05 [0.42–2.64], *p* = 0.92
BMI (kg/m^2^)	26.3 ± 4.4	28.3 ± 4.8	0.073	HR 1.08 [0.99–1.17], *p* = 0.1
Coronary Heart Disease	28 (36.4)	6 (25)	0.445	HR 1.55 [0.56–4.3], *p* = 0.4
Arterial Hypertension	17 (22.1)	7 (29.2)	0.567	HR 0.8 [0.31–2.05], *p* = 0.64
Diabetes mellitus	15 (19.5)	6 (25)	0.569	HR 0.59 [0.21–1.63], *p* = 0.31
ICD	64 (83.1)	21 (87.5)	0.647	HR 0.77 [0.23–2.61], *p* = 0.68
CRT	34 (44.2)	13 (54.2)	0.396	HR 0.71 [0.32–1.6], *p* = 0.41
GFR (mL/min/1.73m^2^)	59.3 ± 27.3	47.9 ± 26.3	0.074	HR 0.98 [0.97–1.002], *p* = 0.081
Smoker	18 (23.3)	13 (54.2)	0.078	HR 2.91 [1.14–7.41], *p* = 0.03
NYHA	3.5 [3–4]	4 [3–4]	0.365	HR 1.56 [0.68–3.61], *p* = 0.3
LVEF (%)	25.8 ± 5.7	26.5 ± 6.1	0.638	HR 0.98 [0.93–1.02], *p* = 0.31
LA Diameter (mm)	47.7 ± 10.1	49.3 ± 6.1	0.621	HR 1.02 [0.96–1.08], *p* = 0.55
LVEDD (mm)	62.5 ± 11.8	63 ± 10.0	0.859	HR 1 [0.97–1.04], *p* = 0.98
AHI	15.7 ± 14.7	16.4 ± 13.0	0.844	HR 1 [0.97–1.03], *p* = 0.98
oAHI	10.7 ± 24.7	8.1 ± 15.2	0.698	HR 0.99 [0.97–1.02], *p* = 0.68
cAHI	12.6 ± 23.7	17.5 ± 33.7	0.513	HR 1.01 [0.99–1.02], *p* = 0.54
ODI	15.8 ± 15.2	13.9 ± 12.1	0.596	HR 0.99 [0.96–1.02], *p* = 0.53
Mean nocturnal SpO2 (%)	90.8 ± 16.2	93 ± 2.8	0.516	HR 1.02 [0.96–1.07], *p* = 0.57
Lowest nocturnal SpO2 (%)	78.5 ± 15.9	78.1 ± 10.3	0.903	HR 1.001 [0.97–1.03], *p* = 0.95
T90 (min)	33.4 ± 65.9	94.7 ± 105.6	0.001	HR 1.007 [1.004–1.011], *p* < 0.001
T90 ≥ 180 m min.	3 (3.9)	4 (16.7)	0.032	HR 4.18 [1.42–12.27], *p* = 0.009
Systolic Blood Pressure (mmHg)	112.2 ± 17.5	101.7 ± 15.3	0.037	HR 0.96 [0.932–0.998], *p* = 0.04
Diastolic Blood Pressure (mmHg)	72.9 ± 11.6	71.6 ± 11.3	0.707	HR 0.98 [0.93–1.04], *p* = 0.56

*Note:* Data are presented as mean ± standard deviation, median [interquartile range], or *n* (%). *p* values were calculated using *t*‐tests, Mann–Whitney *U* tests, or chi‐square tests as appropriate.

Abbreviations: AHI, apnea‐hypopnea index; BMI, body mass index; cAHI, central apnea‐hypopnea index; CRT, cardiac resynchronization therapy; GFR, glomerular filtration rate; ICD, implantable cardioverter‐defibrillator; LA, left atrial; LVEF, left ventricular ejection fraction; LVEDD, left ventricular end‐diastolic diameter; LVAD, left ventricular assist device; NYHA, New York Heart Association; oAHI, obstructive apnea‐hypopnea index; ODI, oxygen desaturation index; *T* < 90, time with oxygen saturation below 90%.

Overall, the cohort presented with severe SDB (mean AHI: 15.9 ± 14.3), which was predominantly central in nature (mean cAHI: 13.6 ± 25.8 vs. mean oAHI: 10.2 ± 23.1). As shown in Tables 1 and 2, the baseline univariate analysis identified nocturnal hypoxemic burden (t90), systolic blood pressure, and smoking as significant predictors of the 5‐year endpoint. In contrast, traditional SDB metrics, including AHI, cAHI, oAHI, ODI, mean nocturnal SpO2, and nadir SpO2, were not associated with outcomes.

**FIGURE 1:**
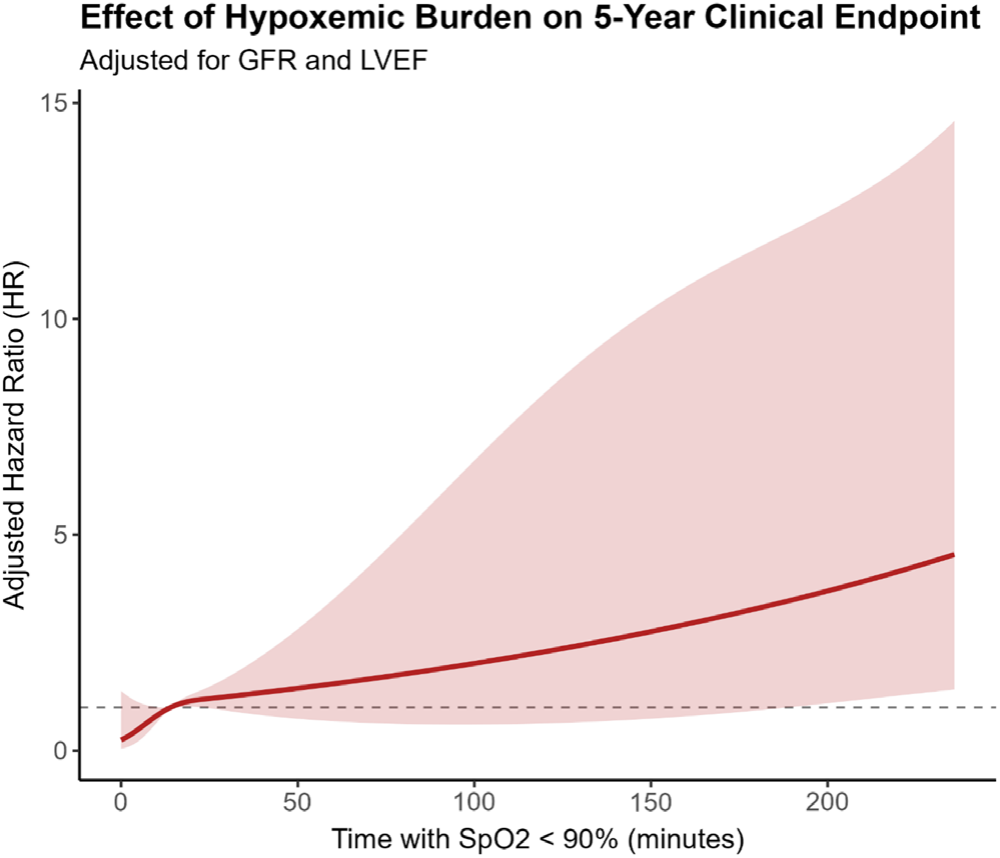
Non‐linear relationship between hypoxemic burden and the 5‐year clinical endpoint. The plot displays the adjusted Hazard Ratio (HR) and 95% confidence interval (shaded area) for the primary endpoint across the continuous range of time spent with SpO2 < 90% (t90). Results are derived from a multivariable Cox proportional hazards model, adjusted for glomerular filtration rate (GFR) and left ventricular ejection fraction (LVEF). The dashed line at HR = 1.0 indicates the line of no effect. The analysis demonstrates a threshold effect, where the risk becomes statistically significant for t90 values exceeding approximately 180 min.

The primary prognostic analysis focused on nocturnal hypoxemic burden (t90). When analysed as a continuous variable, t90 demonstrated a significant, non‐linear dose–response relationship with the 5‐year endpoint (Figure 1). In the final multivariable model, adjusted for GFR and LVEF, each 30‐min increase in t90 remained a powerful independent predictor, associated with a 29% increase in hazard (Adjusted HR: 1.29, 95% CI: 1.14–1.47, *p* < 0.001).

**FIGURE 2:**
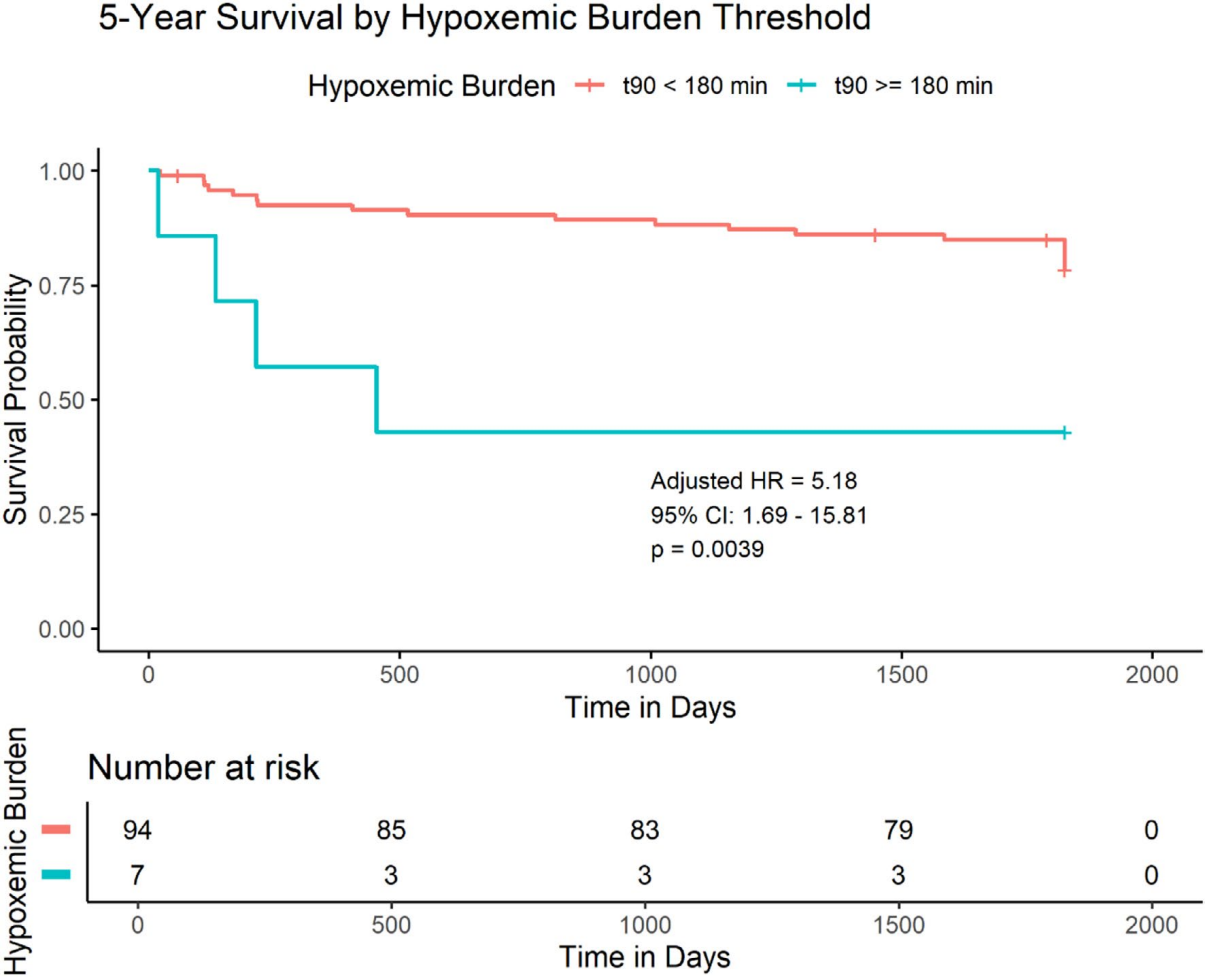
Kaplan–Meier survival curves for the 5‐year primary endpoint, stratified by a hypoxemic burden (t90) of 180 min. The inset text displays the Hazard Ratio (HR), 95% Confidence Interval (CI), and *p* value from the multivariable Cox model, adjusted for GFR and LVEF. An unadjusted log‐rank test also confirmed a significant difference between the curves (*p* = 0.0043).

The spline analysis (Figure 1) identified a clinical threshold around 180 min where the risk became statistically significant. To illustrate this, patients were stratified into high‐ and low‐burden groups. Kaplan–Meier analysis showed a markedly lower 5‐year survival in the high‐burden group (*T* < 90 ≥ 180 min), a difference that was highly statistically significant (*Visual Abstract*; log‐rank *p* = 0.0043). In the final multivariable Cox model, a *T* < 90 of 180 min or more remained a strong and independent predictor of the primary endpoint, conferring a more than five‐fold increase in hazard (Figure 2; Adjusted HR: 5.18, 95% CI: 1.69–15.81, *p* = 0.0039).

## 4 Discussion

In this prospective study of end‐stage HF patients evaluated for HTX, we found that nocturnal hypoxemic burden is a powerful and independent predictor of the 5‐year composite endpoint of death or LVAD implantation. Our primary analysis identified a strong, independent dose–response relationship between continuous t90 and adverse outcomes (Adjusted HR per 30 min: 1.29, *p* < 0.001). This analysis also identified a clinical threshold of approximately 180 min of t90, above which the risk increased more than fivefold after adjusting for key confounders.

Our findings align with and extend a growing body of evidence suggesting that the AHI is an insufficient metric for risk stratification in HF (Oldenburg et al. 2016). Our own comprehensive univariate analysis strongly supports this; while t90 was a powerful predictor, traditional metrics including AHI, cAHI, oAHI, ODI, mean SpO2, and nadir SpO2 all failed to show a significant association with the 5‐year endpoint. In advanced HF, circulatory delay can lengthen respiratory events, paradoxically lowering the AHI even as the severity of SDB worsens (Oldenburg et al. 2016). This has led researchers to focus on hypoxemic burden as a more direct measure of physiological stress. Landmark studies by Oldenburg et al. and Gellen et al. have previously demonstrated in large HF cohorts that t90, not AHI, was the most robust predictor of mortality (Oldenburg et al. 2016; Gellen et al. 2016). Our study is one of the first to specifically validate the profound prognostic importance of t90 in the unique, high‐stakes population of patients actively listed for HTX.

Furthermore, recent work highlights that nocturnal hypoxemic burden is complex, potentially comprising distinct components. Pinna et al. distinguished between respiratory event‐related and non‐specific contributions to T90 in HFrEF, finding that only the non‐specific component retained independent prognostic significance after adjustment (Pinna et al. 2024). The overall prognostic importance of T90 also extends beyond HFrEF, as Mourmans et al. recently demonstrated its independent association with adverse outcomes in patients with HF with preserved ejection fraction (HFpEF) (Mourmans et al. 2025). Understanding these components may have therapeutic implications. Baumert et al. showed that while adaptive servo‐ventilation (ASV) effectively reduces the apnea‐related hypoxemic burden in HFrEF, a significant non‐specific burden often persists (Baumert et al. 2023). This residual hypoxemia might partly explain the lack of mortality benefit, and indeed the excess mortality, observed in large ASV trials like SERVE‐HF (Cowie et al. 2015). This reinforces the critical question raised by our findings: whether targeted interventions to reduce a patient's total t90 can improve survival on the transplant waiting list.

The primary clinical implication of our study is that routine screening for SDB should be strongly considered for all patients being evaluated for HTX. Crucially, this screening should focus on quantifying the total hypoxemic burden (t90) rather than relying solely on the AHI. The identification of a high‐risk continuous burden or a threshold around 180 min, may help clinicians identify a high‐risk subgroup on the waitlist who could warrant closer monitoring or earlier consideration for advanced therapies. The high prevalence of predominantly central sleep apnea in our cohort further underscores that the underlying mechanism in these patients is complex and tied to the pathophysiology of HF itself, not just obesity.

This study has several limitations. As a single‐centre study, our findings require validation in larger, multi‐centre cohorts. Second, our final enrolled cohort of 101 patients was smaller than the *N* = 500 originally planned for the study's initial mechanistic pre‐post design, a change necessitated by slower‐than‐anticipated recruitment and a desire to avoid cohort heterogeneity during a prolonged enrolment. This modest sample size limited the statistical power for some sub‐analyses; for instance, the number of patients in the highest risk group (t90 ≥ 180 min) was small, though the effect size was large and statistically significant, which is reflected in the wide confidence interval for this specific threshold. However, we must emphasise that the study was robustly powered for its primary finding: a post hoc power calculation confirmed our cohort had approximately 89.3% power to detect the significant, continuous dose–response relationship between t90 and the 5‐year endpoint. Furthermore, a significant amount of data for follow‐up sleep studies after transplantation was missing, which precluded a formal analysis of changes in SDB post‐HTX and prompted our deliberate pivot to this prognostic analysis using the complete baseline and follow‐up data.

**TABLE 2:** Unadjusted and multivariable cox proportional hazards analysis for the 5‐year endpoint.

Variable	Model 1 (unadjusted)	Model 2 (adjusted)
	HR (95% CI), *p*	HR (95% CI), *p*
T90 ≥ 180 min	4.18 (1.42–12.27), 0.009	5.18 (1.69–15.81), 0.004
GFR		0.98 (0.96–1), 0.052
LVEF		0.97 (0.92–1.02), 0.250

*Note:* Data are presented as Hazard Ratio (95% Confidence Interval) and *p* value.

Abbreviations: CI, confidence interval; GFR, glomerular filtration rate; HR, hazard ratio; LVEF, left ventricular ejection fraction; T90, time with oxygen saturation below 90%.

In conclusion, nocturnal hypoxemic burden is a powerful and independent predictor of adverse outcomes in patients with end‐stage HF evaluated for HTX. Whether targeted interventions to reduce a patient's t90 can improve survival on the transplant waiting list is a critical question that warrants investigation in future clinical trials.

## Author Contributions

**Max Potratz:** data clearance, data Analysis, manuscript draft and revision. **Tatevik Manukyan:** data collection, manuscript draft and Revision. **René Schramm:** data collection, manuscript revision. **Angelika Costard‐Jäckle:** study and data management. **Uwe Fuchs:** data collection, manuscript draft. **Michiel Morshuis:** study conduct, preparation of the manuscript. **Sabina P. W. Guenther:** data collection, manuscript draft. **Sebastian V Rojas:** study conduct, study overview, data and manuscript administration. **Volker Rudolph:** study conduct, study overview, data and manuscript administration. **Jan F. Gummert:** study conduct, study overview, data and manuscript administration. **Henrik Fox:** principal investigator, study planning, data collection, results and discussion, manuscript draft, revision, final approval.

## Funding

This work was supported by Deutsche Stiftung für Herzforschung, F/14/16 (German Heart Foundation/German Foundation of Heart Research).

## Conflicts of Interest

The authors declare no conflicts of interest.

## Data Availability

The data that support the findings of this study are available on request from the corresponding author. The data are not publicly available due to privacy or ethical restrictions.

## References

[jsr70256-bib-0001] Baumert, M. , D. Linz , M. Pfeifer , et al. 2023. “Hypoxaemic Burden in Heart Failure Patients Receiving Adaptive Servo‐Ventilation.” ESC Heart Failure 10, no. 6: 3725–3728. 37794711 10.1002/ehf2.14556 PMC10682887

[jsr70256-bib-0002] Cowie, M. R. , H. Woehrle , K. Wegscheider , et al. 2015. “Adaptive Servo‐Ventilation for Central Sleep Apnea in Systolic Heart Failure.” New England Journal of Medicine 373, no. 12: 1095–1105. 26323938 10.1056/NEJMoa1506459 PMC4779593

[jsr70256-bib-0003] Fox, H. , H.‐C. Purucker , I. Holzhacker , et al. 2016. “Prevalence of Sleep‐Disordered Breathing and Patient Characteristics in a Coronary Artery Disease Cohort Undergoing Cardiovascular Rehabilitation.” Journal of Cardiopulmonary Rehabilitation and Prevention 36, no. 6: 421–429. 27490427 10.1097/HCR.0000000000000192

[jsr70256-bib-0004] Gellen, B. , F. Canouï‐Poitrine , L. Boyer , et al. 2016. “Apnea‐Hypopnea and Desaturations in Heart Failure With Reduced Ejection Fraction: Are We Aiming at the Right Target?” International Journal of Cardiology 203: 1022–1028. 26630630 10.1016/j.ijcard.2015.11.108

[jsr70256-bib-0005] Gottlieb, J. D. , A. R. Schwartz , J. Marshall , et al. 2009. “Hypoxia, Not the Frequency of Sleep Apnea, Induces Acute Hemodynamic Stress in Patients With Chronic Heart Failure.” Journal of the American College of Cardiology 54, no. 18: 1706–1712. 19850211 10.1016/j.jacc.2009.08.016 PMC2808691

[jsr70256-bib-0006] Heidenreich, P. A. , B. Bozkurt , D. Aguilar , et al. 2022. “2022 AHA/ACC/HFSA Guideline for the Management of Heart Failure: A Report of the American College of Cardiology/American Heart Association Joint Committee on Clinical Practice Guidelines.” Circulation 145, no. 18: e895–e1032. 35363499 10.1161/CIR.0000000000001063

[jsr70256-bib-0007] Kapur, V. K. , D. H. Auckley , S. Chowdhuri , et al. 2017. “Clinical Practice Guideline for Diagnostic Testing for Adult Obstructive Sleep Apnea: An American Academy of Sleep Medicine Clinical Practice Guideline.” Journal of Clinical Sleep Medicine 13, no. 3: 479–504. 28162150 10.5664/jcsm.6506 PMC5337595

[jsr70256-bib-0008] Levy, D. , S. Kenchaiah , M. G. Larson , et al. 2002. “Long‐Term Trends in the Incidence of and Survival With Heart Failure.” New England Journal of Medicine 347, no. 18: 1397–1402. 12409541 10.1056/NEJMoa020265

[jsr70256-bib-0009] McDonagh, T. A. , M. Metra , M. Adamo , et al. 2021. “2021 ESC Guidelines for the Diagnosis and Treatment of Acute and Chronic Heart Failure.” European Heart Journal 42, no. 36: 3599–3726. 34447992 10.1093/eurheartj/ehab368

[jsr70256-bib-0010] Mourmans, S. G. J. , J. Weerts , M. Baumert , et al. 2025. “Prognostic Value of Hypoxaemic Burden From Overnight Oximetry in Heart Failure With Preserved Ejection Fraction.” ESC Heart Failure 12, no. 1: 622–630. 39462183 10.1002/ehf2.15116 PMC11769663

[jsr70256-bib-0011] Oldenburg, O. , B. Wellmann , A. Buchholz , et al. 2016. “Nocturnal Hypoxaemia Is Associated With Increased Mortality in Stable Heart Failure Patients.” European Heart Journal 37, no. 21: 1695–1703. 26612581 10.1093/eurheartj/ehv624

[jsr70256-bib-0012] Pinna, G. D. , R. Maestri , E. Robbi , G. Guazzotti , A. Caporotondi , and M. T. La Rovere . 2024. “Nocturnal Hypoxemic Burden in Patients With Heart Failure: Emerging Prognostic Role of Its Nonspecific Component.” American Heart Journal 276: 1–11. 38972337 10.1016/j.ahj.2024.06.011

[jsr70256-bib-0013] Sharma, S. , R. Stansbury , B. Hackett , and H. Fox . 2021. “Sleep Apnea and Pulmonary Hypertension: A Riddle Waiting to Be Solved.” Pharmacology & Therapeutics 227: 107935. 34171327 10.1016/j.pharmthera.2021.107935

[jsr70256-bib-0014] Sohns, C. , H. Fox , N. F. Marrouche , et al. 2023. “Catheter Ablation in End‐Stage Heart Failure With Atrial Fibrillation.” New England Journal of Medicine 389, no. 15: 1380–1389. 37634135 10.1056/NEJMoa2306037

[jsr70256-bib-0015] Sohns, C. , N. F. Marrouche , A. Costard‐Jäckle , et al. 2021. “Catheter Ablation for Atrial Fibrillation in Patients With End‐Stage Heart Failure and Eligibility for Heart Transplantation.” ESC Heart Failure 8, no. 2: 1666–1674. 33314690 10.1002/ehf2.13150 PMC8006697

